# RIG-I Enhanced Interferon Independent Apoptosis upon Junin Virus Infection

**DOI:** 10.1371/journal.pone.0099610

**Published:** 2014-06-11

**Authors:** Olga A. Kolokoltsova, Ashley M. Grant, Cheng Huang, Jennifer K. Smith, Allison L. Poussard, Bing Tian, Allan R. Brasier, Clarence J. Peters, Chien-Te Kent Tseng, Juan C. de la Torre, Slobodan Paessler

**Affiliations:** 1 Department of Pathology, University of Texas Medical Branch (UTMB), Galveston, Texas, United States of America; 2 Internal Med-Endocrinology, UTMB, Galveston, Texas, United States of America; 3 Department of Microbiology and Immunology, UTMB, Galveston, Texas, United States of America; 4 Department of Immunology and Microbial Science, The Scripps Research Institute, La Jolla, California, United States of America; George Mason University, United States of America

## Abstract

Junin virus (JUNV) is the etiological agent of Argentine hemorrhagic fever (AHF), a human disease with a high case-fatality rate. It is widely accepted that arenaviral infections, including JUNV infections, are generally non-cytopathic. In contrast, here we demonstrated apoptosis induction in human lung epithelial carcinoma (A549), human hepatocarcinoma and Vero cells upon infection with the attenuated Candid#1 strain of, JUNV as determined by phosphatidylserine (PS) translocation, Caspase 3 (CASP3) activation, Poly (ADP-ribose) polymerase (PARP) cleavage and/or chromosomal DNA fragmentation. Moreover, as determined by DNA fragmentation, we found that the pathogenic Romero strain of JUNV was less cytopathic than Candid#1 in human hepatocarcinoma and Vero, but more apoptotic in A549 and Vero E6 cells. Additionally, we found that JUNV-induced apoptosis was enhanced by RIG-I signaling. Consistent with the previously reported role of RIG-I like helicase (RLH) signaling in initiating programmed cell death, we showed that cell death or DNA fragmentation of Candid#1-infected A549 cells was decreased upon siRNA or shRNA silencing of components of RIG-I pathway in spite of increased virus production. Similarly, we observed decreased DNA fragmentation in JUNV-infected human hepatocarcinoma cells deficient for RIG-I when compared with that of RIG-I-competent cells. In addition, DNA fragmentation detected upon Candid#1 infection of type I interferon (IFN)-deficient Vero cells suggested a type I IFN-independent mechanism of apoptosis induction in response to JUNV. Our work demonstrated for the first time apoptosis induction in various cells of mammalian origin in response to JUNV infection and partial mechanism of this cell death.

## Introduction

Arenaviruses are bisegmented, negative sense RNA viruses with enveloped virions that use an ambisense coding strategy [1]. The large segment of the arenavirus genome encodes for a RNA-dependent RNA polymerase (L) with endonuclease cap snatching activity [2] and a small RING finger protein (Z) with matrix-like functions [3]. The small segment encodes for the viral nucleoprotein (NP), endowed with a 3′ to 5′ exoribonuclease activity [4], and the glycoprotein precursor (GPC). Co-translational cleavage of GPC by the cellular signal peptidase produces a 58 amino acid stable signal peptide (SSP), and subsequent posttranslational cleavage by the cellular site 1 protease produces peripheral virion attachment (GP1) and fusion-active transmembrane (GP2) proteins [5]. The members of the *Arenaviridae* family are divided into two serologically and geographically distinct groups: New and Old World arenaviruses [6]. Five members of the New World clade B arenaviruses (Junin, Guanarito, Sabia, Machupo and Chapare), and the Old World Lassa virus (LASV) and Lujo virus [7] can cause severe hemorrhagic fever disease in humans [8].

AHF, caused by JUNV, is characterized by gastrointestinal, cardiovascular, hematological, renal, immunological, neurological and hemorrhagic manifestations [9]. An estimated 3–5 million people in central and northwestern Argentina are at risk of developing the disease [10], [11]. For the last 50 years, the AHF endemic area has increased nearly ten times in size as a result of the expanding geographic distribution of JUNV natural host–drylands vesper mouse [10]. Moreover, the ease of aerosol infection [12], [13], high case-fatality rate (15–42%) [14], [15], and the lack of virus-specific drugs [16], [17] make JUNV a potential candidate for weaponization. Accordingly, JUNV is a National Institute of Allergy and Infectious Diseases (NIAID) Category A Priority Pathogen [18] and is considered a Select Agent by the Centers for Disease Control and Prevention (CDC), U.S. Department of Agriculture (USDA) and U.S. Department of Health and Human Services (HHS) [19], [20].

Cytopathic effect (CPE) *in vitro* has been reported only in response to infections by non-pathogenic arenaviruses. On the other hand, arenaviruses associated with hemorrhagic diseases in humans, including JUNV, are generally considered to be non-cytopathic viruses [21]–[23]. The non-pathogenic clade B arenavirus Tacaribe, but not the pathogenic Romero strain of JUNV, was documented to induce pronounced CPE in Vero cells and conglomeration of human blood purified monocytes [22]. Exposure of PS, a phospholipid component kept on the inner-leaflet of cell membranes in normal cells, on the surface of transformed mouse monocytes/macrophages infected with Pichinde virus (a New World arenavirus that is non-pathogenic for humans) has been described [24]. A recent report [23] documented an absence of apoptosis induction in Vero E6 cells infected with the Romero strain of JUNV. The lack of apoptosis in these cells was proposed to be mediated by the caspase decoy function of Romero NP [23].

A safe and effective live-attenuated JUNV vaccine (Candid#1) is licensed in Argentina and has been used with success within the JUNV endemic area to prevent AHF [25]. However, the documented genetic and virulence heterogeneity of Candid#1 [26], and the lack of understanding of the mechanisms underlying Candid#1 attenuation pose great barriers to its acceptance in the United States. Compared with its parental, as well as with other virulent JUNV strains, Candid#1 contains multiple amino acid changes in GP, NP and L that hinder the identification of the genetic markers of attenuation [27]–[32].

We have recently documented an induction of type I IFN in response to pathogenic, Romero, and attenuated, Candid#1, strains of JUNV infection in human lung epithelium carcinoma cells (A549) [33]. We also showed that siRNA-mediated down-regulation of RIG-I or IRF3 production in A549 cells resulted in drastic reduction of STAT1 phosphorylation and IFN-stimulated gene (ISG) induction in response to Candid#1 infection. These data revealed RIG-I as a primary trigger of type I IFN signaling in response to JUNV infection in A549 cells [33]. We also observed that the ISG response was substantially reduced at the mRNA level in A549 cells infected with the virulent Romero strain compared with that of Cadid#1-infected cells, indicating that Candid#1 might be a more potent stimulator of the RIG-I/IRF3 signaling pathway than Romero. However, both viruses were resistant to the antiviral effect of type I or II IFN pre-treatment in Vero cells indicating that the attenuation of Candid#1 is not related to a higher susceptibility to the antiviral status induced by these cytokines [33].

Initiation of apoptosis in response to viral infection [34] has been linked to RLH signaling independent of type I IFN pathway activation [35]–[39]. Specifically, IFN-I-independent, caspase-9, Apaf-1-dependent signaling trough RIG-I/MDA5-MAVS-mediated induction of Puma and Noxa transcription [36], [40]; or MAVS mediated IFN-I, IRF3, NF-ΚB-independent, caspase-3, -9-dependent [35]; or IFN-I, NF-ΚB-independent, RIG-I-TRAF3/TRAF2/TRAF6–mediated IRF3 interaction with Bax protein [38], [39] has been shown to activate mitochondrial apoptotic pathway in response to dsRNA or viral infection. We observed that infection with Candid#1 JUNV induces CPE in primate cell lines. These findings led us to hypothesize that Candid#1 infection induces a RLH-mediated type I IFN-independent apoptosis. In this study we have used different experimental approaches to test our hypothesis. Here we demonstrated apoptosis induction in two human cell lines and Vero cells upon infection with the attenuated Candid#1 vaccine strain of JUNV as determined by PS translocation, CASP3 activation, PARP cleavage and/or chromosome DNA fragmentation. Furthermore, to investigate the potential role of apoptosis induction in Candid#1 attenuation or JUNV pathogenesis we assayed cell death induction in response to the pathogenic Romero as well. We found that the pathogenic Romero strain of JUNV was less cytopathic than Candid#1 in human hepatocarcinoma and Vero cells as determined by DNA fragmentation. However, stronger apoptosis induction was observed in A549 and Vero E6 cells in response to Romero infection. Additionally, we found that JUNV-induced apoptosis was enhanced by RIG-I signaling but independent of production of type I IFN.

## Materials and Methods

### Cell Lines

Vero [41], Vero E6 [42] and human lung epithelial carcinoma (A549) cells (American Tissue Culture Collection, Manassas, VA) were maintained in minimum essential medium (MEM) or F-12K medium Kaighn’s modification supplemented with 10% fetal bovine serum and 1% Penicillin-Streptomycin (10,000 U/mL). Huh 7 and Huh 7.5 cells [43] (kindly provided by Dr. Charles Rice, New-York, NY) were maintained in Dulbecco’s Modified Eagle Medium (DMEM) supplemented with 10% fetal bovine serum and 1% Penicillin-Streptomycin (10,000 U/mL).

### Viruses

Romero (GenBank accession nos. AY619640 and AY619641) [44] and Candid#1 (GenBank accession no. U70801) strains of JUNV were obtained from Dr. Thomas G. Ksiazek (Centers for Disease Control and Prevention, Atlanta, GA) and Dr. Robert Tesh (The World Reference Center for Emerging Viruses and Arboviruses (WRCEVA), University of Texas Medical Branch, Galveston, TX), respectively. Virus stocks were prepared using Vero cells. Cell debris in supernatants were filtered out through 0.45 µm HV Durapore Membrane Stericup sterile vacuum filtration system (Millipore Corporation, Billerica, MA). Cleared supernatants were concentrated through 30 min centrifugation at 3220×g using Amicon Ultra-15 Centrifugal Filter Unit PLHK Ultracel-PL Membrane, 100 kDa (Millipore Corporation, Billerica, MA). All work with JUNV Romero was performed at the University of Texas Medical Branch BSL-4 facilities (Robert E. Shope Laboratory or the Galveston National Laboratory) in accordance with institutional health and safety guidelines and federal regulations [45].

### Plaque Assay on Vero Cells

Cells were plated in 12-well multiwell plates. Ten-fold supernatant dilutions were added to cell monolayers for 1 h at the 37°C, 5% CO_2_. Following incubation, cells were overlaid with MEM containing 0.6% gum tragacanth, 1% fetal bovine serum, and 1% Penicillin-Streptomycin (10,000 U/mL). Following 8 days incubation, cells were fixed with 10% formaldehyde and stained with crystal violet.

### Cell Viability Determination

Cells (1×10^4^) were mock-infected or infected with JUNV in quadruplicates or triplicates. Cell viability was assayed using Cell Growth/Viability Determination Kit MTT Based (Sigma-Aldrich, St. Louis, MO) according to the manufacturer’s instruction.

### Apoptosis Detection

A549 cells (1.2×10^5^) were mock-infected or infected with JUNV in triplicates. Cells were stained using FITC Annexin V Apoptosis Detection Kit I (BD Biosciences, San Jose, CA) and LIVE/DEAD Fixable Far Red Dead Cell Stain Kit (Molecular Probes, Inc./Invitrogen, OR) per manufacturers’ instructions. Staurosporine (Sigma-Aldrich, St. Louis, MO) was used as a positive control to induce apoptosis at 1 µM for 3 to 5 h. Stained cells were analyzed using BD FACSCanto (BD Biosciences, San Jose, CA) and FlowJo 7.6.5 (Tree Star, Inc., Ashland, OR). FACS analysis was conducted at the Flow Cytometry and Cell Sorting Core Facility, UTMB.

For assessment of mono-and oligonucleosomes, cells (1×10^4^) were mock-infected or infected with JUNV in triplicates, and analyzed with the Cell Death Detection ELISA (Roche Applied Science, Indianapolis, IN) according to the manufacturer’s instruction.

Treatment with camptothecin (Sigma-Aldrich, St. Louis, MO) at 20 µM for 16 h was included as a positive control of apoptosis induction for the detection of cleaved CASP3 and PARP.

### Down-regulation of Gene Expression via siRNA

ON-TARGET plus SMART pool siRNA targeting human DDX58, IRF3 or Non-targeting Pool (Thermo Fisher Scientific Inc, Pittsburgh, PA) were transfected into A549 cells by electroporation using Amaxa Cell Line Nucleofector Kit T (Lonza Walkersville, Inc., Walkersville, MD) according to the manufacturer’s protocol. At 24 h post transfection, cells were seeded into 12-well plates. At 1.5 days post transfection cells were mock-infected or infected with Candid#1.

### Poly(I:C) Transfection

Cell lysates were collected 16 h post mock- or Poly(I:C)-LMW/LyoVec (InvivoGen, San Diego, CA) transfection (4 µg/mL) per manufacturers’ instructions.

### Western Blotting

Cell lysates were collected in 1×RIPA buffer (Cell Signaling Technology, Inc., Danvers, MA) supplemented with Complete Mini, EDTA-free protease inhibitor (Roche Applied Science, Indianapolis, IN). Protein concentration was assayed by Bio-Rad Protein Assay (Bio-Rad, Hercules, CA). Proteins were resolved on 4–20% SDS-PAGE gel and transferred to PVDF membrane using Mini Trans-Blot Electrophoretic Transfer Cell apparatus (Bio-Rad, Hercules, CA). Primary antibodies used for western blot analysis were rabbit monoclonal RIG-I (D14G6), cleaved CASP3 (Asp175) (5A1E), cleaved PARP (Asp214) (D64E10) XP (Cell Signaling technology, Inc., Danvers, MA), IRF3 antibody [EP2419Y] (Abcam, Cambridge, MA), and goat polyclonal anti-actin (I-19) antibody (Santa Cruz Biotechnology, Inc., Santa Cruz, CA). Secondary antibodies used were HRP-linked goat anti-rabbit IgG (Cell Signaling technology, Inc., Danvers, MA) and HRP-conjugated donkey anti-goat IgG (Santa Cruz Biotechnology, Inc., Santa Cruz, CA). Immune complexes were visualized with Amersham ECL Western Blotting Detection Reagents and exposed to X-ray films (GE Healthcare Bio-Sciences Corp., Piscataway, NJ) according to the manufacturer’s instruction. The protein level was quantitated by densitometry measurement using AlphaEaseFC software (Alpha Innotech, Miami, FL).

### Statistical Analysis

Data were analyzed by two-way or three- way ANOVA using SigmaPlot 12.0 (Systat Software, Inc., San Jose, CA).

## Results

### A549 Cells Undergo Apoptosis during Candid#1 Infection

**Figure 1:**
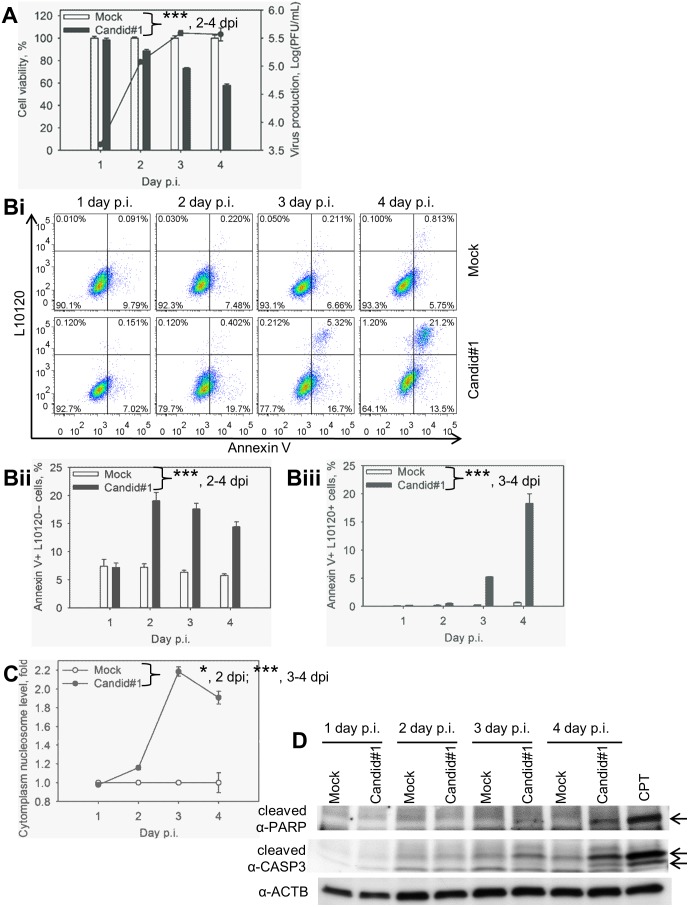
Candid#1 JUNV infection induces apoptosis in A549 cells. Cells were mock-infected (Mock) or infected (MOI = 1.75 PFU/cell) with Candid#1. **A.** Reduction of cell viability in infected cells correlated with virus production. Production of infectious progeny and cell viability determined using plaque assay and MTT-based assay, respectively. Cell viability of mock-infected samples was assigned as 100%. **B.** Quantification of PS flipping. Cells were stained with FITC-Annexin V (Annexin V) and the viability dye L10120 and analyzed by FACS. **Bi.** Representative dot-plots. Annexin+L10120−cells (low right quadrant) represent the population of cells at early apoptotic stage. Annexin+L10120+cells (top right quadrant) represent apoptotic and dead cells. **Bii.** Percentage of cells at early stage of apoptosis. **Biii.** Percentage of apoptotic and dead cells. **C.** Accumulation of mono- and oligonucleosomes in the cytoplasm was determined by ELISA. For **A, Bii, Biii** and **C**, data represent the average of three replicates ±SEM. *** and * - P. value<0.001 or <0.05, respectively. **D.** Activation of CASP3 and PARP cleavage in infected cells. Cell lysates were subjected to western blotting for β-actin- (α-ACTB), CASP3 (α-cleaved CASP3) and PARP (α-cleaved PARP) cleavage. Arrows point to the cleaved form of PARP and CASP3. Camptothecin (CPT) was used as a positive control for apoptosis induction.

A549 cells were mock-infected or infected with Candid#1 and cell viability was determined by an MTT-based viability assay. Viability of Candid#1-infected cells was decreased compared with that of mock-infected controls starting from 2 days p.i. and reached over 42% reduction by day 4 p.i. (Fig. 1A). The highest level of Candid#1-induced reduction in cell viability correlated with a peak production of infectious virus progeny as determined by plaque assay (Fig. 1A).

Next we examined whether the reduced survival observed in Candid#1-infected cells was caused by apoptotic cell death. For this we assessed PS flipping from the inner to the outer layers of cell membrane, which was detected via Annexin V binding. In addition, we examined cell membrane integrity by staining with an amine reactive L10120 viability dye. Stained cells were analyzed by FACS. Transition from early apoptotic (Annexin V+ and L10120−) to late apoptotic state (Annexin V+ and L10120+) is characteristic of apoptotic cell death [46]. In mock-infected samples, the percentages of early (Fig. 1Bi, ii) and late (Fig. 1Bi, iii) apoptotic cells remained largely unchanged from day 1 to day 4 p.i. and were 6.7% and 0.3% on average, respectively. Induction of early apoptosis in Candid#1 infected cells was detected 2 days p.i. (Fig. 1Bi, ii). Likewise, the percentage of late apoptotic cells in Candid#1-infected samples increased from 5.2% at day 3 p.i. to 18.3% at 4 days p.i. (Fig. 1Bi, iii).

Fragmentation of chromosomal DNA (mono- and oligo-nucleosome formation) occurs at the late stage of apoptosis as a result of DNA cleavage by activated endonucleases [47]. We quantified cytoplasmic histone-associated-DNA-fragments through ELISA. Induction of DNA fragmentation in Candid#1-infected cells was 1.2-, 2.2- and 1.9-fold of that in mock-infected cells at days 2, 3 and 4 p.i., respectively (Fig. 1C), a finding consistent with the reduced cell viability observed in virus infected samples (Fig. 1A).

Consistent with the increased level of Annexin V staining and DNA fragmentation, we also detected in Candid#1-infected cells the large fragment of cleaved executioner CASP3 and cleaved PARP, a CASP3 downstream substrate (Fig. 1D) by western blotting using cleaved CASP3 and PARP antibody as described [33]. Treatment with camptothecin was included as a positive control of apoptosis induction.

These data indicate that infection of human lung epithelium carcinoma cells with the attenuated strain of JUNV induces apoptosis.

### RIG-I-enhanced Apoptosis in Response to JUNV Infection

**Figure 2:**
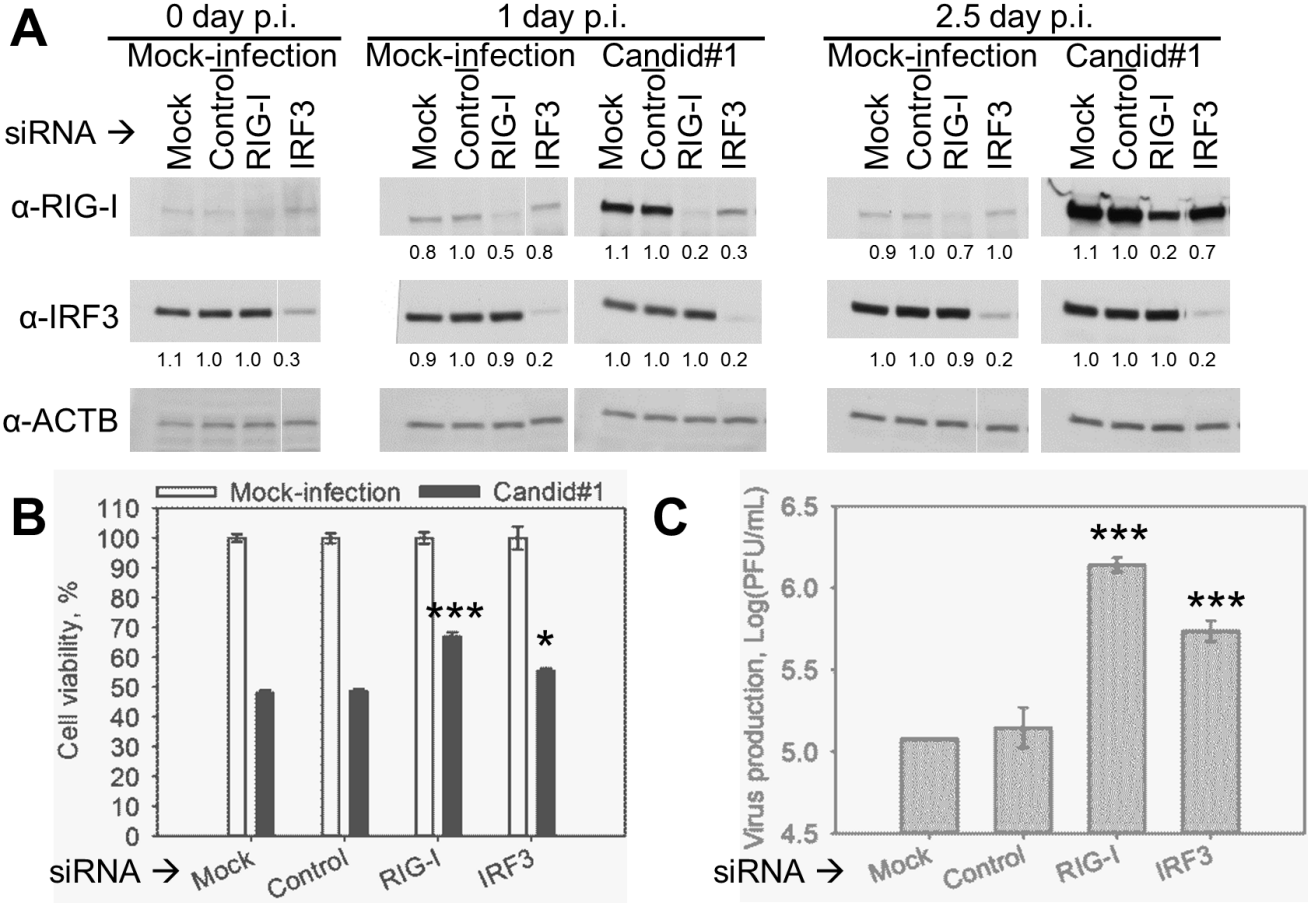
siRNA-mediated down-regulation of RIG-I or IRF3 expression increased cell survival of Candid#1-infected A549 cells. A549 cells were transfected with siRNA targeting RIG-I, IRF3, non-targeting siRNA (Control) or mock-transfected (Mock). At 1.5 days post transfection cells were infected (MOI = 1 PFU/cell) with Candid#1 or mock-infected (Mock-infection). **A.** Down-regulation of RIG-I and IRF3 upon transfection. Cell lysate was collected at 0, 1, and 2.5 days p.i. and subjected to western blotting analysis. Protein level was measured by densitometry analysis and shown as the relative amount as compared with that of Control sample. **B.** Increased viability of infected cells with RIG-I and IRF3 down-regulation. Cell viability at 2.5 days p.i. was evaluated by MTT-based assay. **C.** Increased virus production of infected cells with RIG-I and IRF3 down-regulation. Virus production at 2.5 days p.i. was evaluated by plaque assay. **B–C.** Data represent the average of 3 replicates ±SEM. *** and * indicate P. value<0.001, or <0.05, respectively.

Recently we reported that infection with JUNV, both the pathogenic Romero and live-attenuated Candid#1 vaccine strains, activated the RIG-I/IRF3 signaling pathway as well as IFN-type I signaling in A549 cells [33]. Previously, RLH signaling has been linked to apoptosis induction [35]–[39]. We, therefore, were interested in examining a possible role of RIG-I signaling in apoptosis induction during Candid#1 and Romero infection. For this we first used a siRNA-based approach to down-regulate expression levels of RIG-I and IRF3 in A549 cells as described [33]. Initially we explored the feasibility of this approach using only Candid#1. At 1.5 days post siRNA transfection, cells were mock-infected or infected with Candid#1. Consistent with previous findings [33] both mock-and Candid#1-infected cells transfected with IRF3-specific siRNA exhibited efficient silencing of IRF3 expression (70–80% reduction) at 1 and 2.5 days p.i. as determined by western blotting (Fig. 2A). Similarly to our published data [33], RIG-I expression was induced by Candid#1 at 1 and 2.5 days after infection, since RIG-I is an IFN-stimulated gene. The induction was observed even in the cells transfected with RIG-I-specific siRNA (Figure 2A). Induction of RIG-I expression in response to Candid#1 infection occurred at the time as apoptotic changes became detectable; that made it experimentally difficult to examine the extent of RIG-I contribution to the apoptosis induction. Therefore, we decided not to perform the same experiment with Romero virus in the BSL-4 laboratory. Nevertheless, even the transient knockdown of RIG-I, and to the lesser extent of IRF3, resulted in increased cell viability of Candid#1-infected cells. At 2.5 days p.i. cell viability was 55.4±1.8 and 67.1±2.4% in IRF3 and RIG-I knockdown cells, respectively, versus 48.6±1.4% in Candid#1-infected cells transfected with control siRNA (Fig. 2B). Increased cell survival in RIG-I and IRF3 knockdown Candid#1-infected cells was observed despite 9.3- and 3.7-fold higher virus production, respectively, as compared with that of cells transfected with the control siRNA (Fig. 2C). This observation suggests that the increased cell viability was not due to a reduced viral replication.

**Figure 3:**
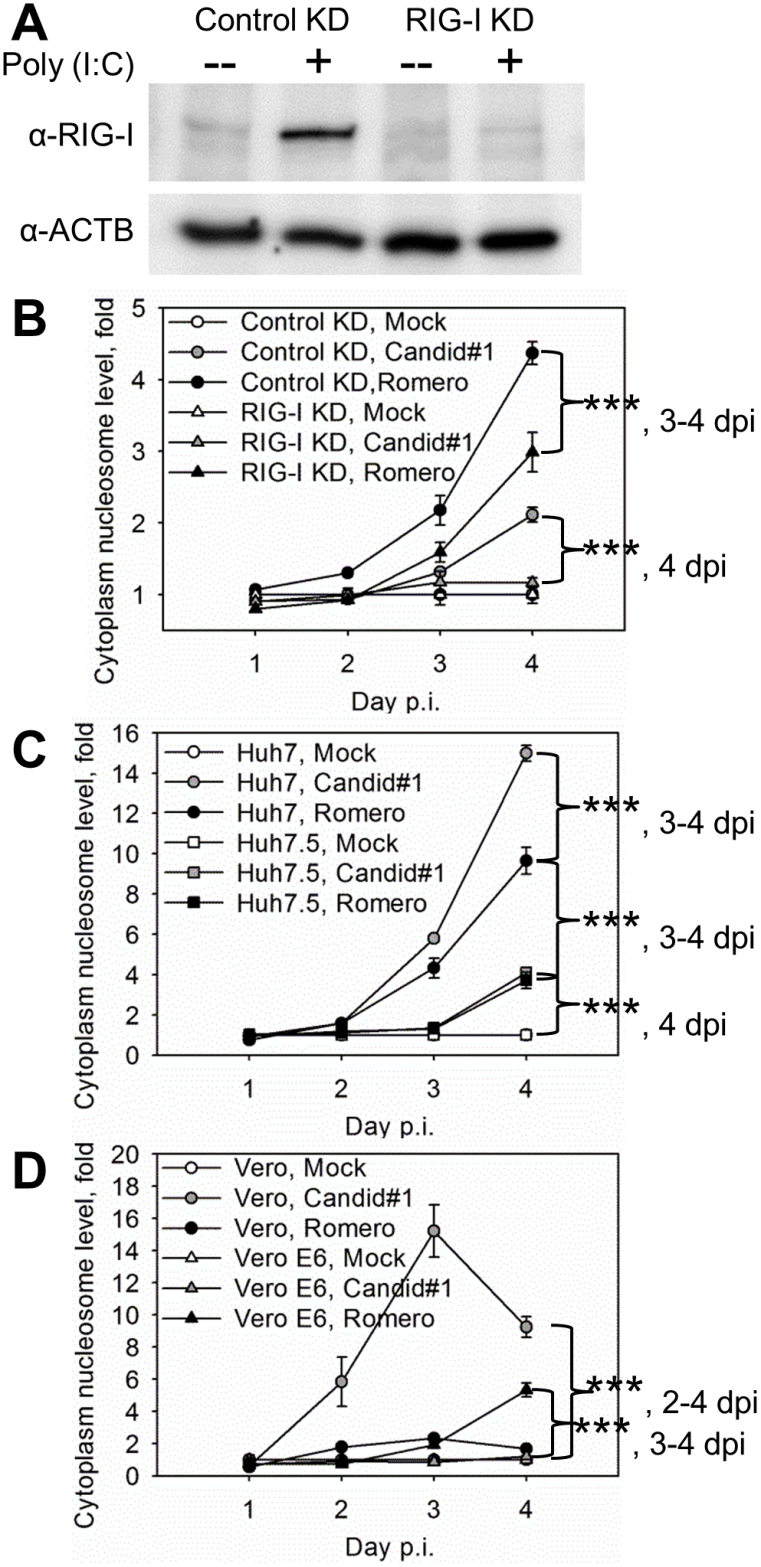
RIG-I-enhanced type I IFN-independent apoptosis in response to JUNV infection. **A–B.** shRNA-mediated stable down-regulation of RIG-I expression diminished DNA fragmentation in infected A549 cells. **A.** Efficient knock-down of RIG-I expression in RIG-I KD cells. Following poly(I:C) transfection RIG-I deficient (RIG-I KD) or competent (Control KD) cells were lysed and subjected to western blotting analysis. **B–D.** Cells were infected (MOI = 1.75 PFU/cell) with Candid#1 or Romero JUNV or mock-infected (Mock). **B.** Diminished DNA fragmentation in infected RIG-I-KD versus that of Control KD cells. **C.** Diminished DNA fragmentation in infected RIG-I-deficient Huh7.5 cells versus that of RIG-I competent Huh7. **D.** Induction of DNA fragmentation in type I IFN-deficient Vero and Vero E6 cells upon Candid#1 or Romero infection, respectively. **B–C.** Accumulation of mono- and oligonucleosomes in the cytoplasm was assayed by ELISA. Data represent the average of 3 replicates ±SEM. *** - P. value<0.001.

Next, we examined whether Romero induced levels of cell apoptosis similar to those observed in Candid#1-infected cells, and whether RIG-I signaling influenced also apoptosis in Romero-infected cells. To achieve a long-term down-regulation of RIG-I expression we generated A549 cells stably transduced with a lentivirus expressing either a RIG-I-targeting shRNA (RIG-I KD) or a control non-targeting shRNA (Control KD). To confirm target knockdown, RIG-I KD and Control KD cells were transfected with Poly(I:C) and cell lysates were examined by western blotting. Induction of RIG-I expression upon poly(I:C) treatment was detected in Control KD but not in RIG-I KD cell lysates (Fig. 3A).

RIG-I KD and Control KD lines were infected with Candid#1 or Romero JUNV or mock-infected, and assessed for cell apoptosis by determining levels of DNA fragmentation. At 4 days p.i. we observed increased levels of DNA fragmentation in Control KD cells infected with either Candid#1 (1.8-fold) or Romero (1.5-fold) compared with RIG-I KD cells infected with the corresponding viruses (Fig. 3B). Reduced DNA fragmentation in the infected RIG-I KD cells was observed despite a 5.8-fold increased production of infectious progeny over that observed in infected Control KD cells at 4 days p.i. (data not shown).

In order to exclude the possibility that the apoptotic response to JUNV infection was cell type-specific, we also mock-infected or infected with Candid#1 or Romero JUNV human hepatocarcinoma cells with a functional (Huh7) or non-functional (Huh7.5) form of RIG-I [43], [48]. DNA fragmentation level was significantly higher in JUNV-infected Huh7 cells than in Huh7.5 cells (4.3- and 3.7-fold for Candid#1 and 3.3- and 2.6-fold for Romero virus) at 3 and 4 days p.i., respectively (Fig. 3C). Production of infectious virus was similar in both cell lines (data not shown).

Together our findings indicate that an active RIG-I signaling pathway enhances apoptosis during JUNV infection of human cells.

### Type I IFN-independent Apoptosis Induction in Response to JUNV Infection

We have shown an induction of type I IFN signaling upon JUNV infection of human cells [33]. Type I IFN signaling has also been linked to induction of apoptosis in response to viral infection [49]. To determine the role of type I IFN in JUNV-induced apoptosis, we analyzed DNA fragmentation and virus production in two type I IFN-deficient cells of non-human primate origin: Vero [41] and its clone Vero E6 [42]. Cells were mock-infected or infected with Candid#1 or Romero. Production of infectious progeny of both Candid#1 and Romero viruses was similar in both cell lines (data not shown). Infection with Candid#1 virus induced cytoplasmic nucleosomes in Vero cells starting at day 2 p.i. Induction of DNA fragmentation in Candid#1-infected Vero cells was 5.8-, 15.2- and 9.2-fold higher than that in mock-infected cells at days 2, 3 and 4 p.i., respectively. Romero infection of Vero E6 cells led to detectable cytoplasmic nucleosome formation (1.9- and 5.3-fold of that in mock-infected Vero E6 cells at days 3 and 4 p.i., respectively). Though, DNA fragmentation in these cells was delayed (3–4 days p.i.) and reduced, relative to that of Candid#1 infection of Vero cells (Fig. 3D). Detection of increased levels of cytoplasmic nucleosomes in type I IFN-deficient Vero cells upon Candid#1 and VeroE6 cells upon Romero infection suggests that type I IFN production is not required for JUNV-induced apoptosis. In contrast, no DNA fragmentation was detected in Romero-infected Vero cells or Candid#1-infected Vero E6 cells (Fig. 3D).

## Discussion

CPE *in vitro* has been documented previously in mammalian cells infected with non-pathogenic arenaviruses Tacaribe [22] and Pichinde [24]. In agreement with that in the current study we have demonstrated an induction of cell death in human cells upon infection with vaccine strain of JUNV, Candid#1. Moreover, for the first time, we confirmed the apoptotic nature of this cell death using four different experimental approaches: 1) PS flipping (detected via Annexin V binding) from the inner to the outer membrane layer of cell that at the same time exclude viability dye is indicative of early apoptosis; 2) Transition over time from early (Annexin V positive, viability dye negative) to late apoptotic state (Annexin V and viability dye positive) provides confirmation of cell death via the apoptotic pathway [46]; 3) Fragmentation of cell DNA as a result of nuclease activation, which is a hallmark of late apoptosis [47], results in mono- and oligo-nucleosome formation that can be quantified using ELISA and 4) Detection of the cleaved CASP3 and PARP is a well-accepted surrogate of activation of the apoptotic cell death pathway. Together these multiple measurements strongly suggest that JUNV Candid#1 infection induces cellular apoptosis. In addition, we detected DNA fragmentation in human hepatocarcinoma and non-human primate Vero cells infected with Candid#1 JUNV.

Moreover, we observed DNA fragmentation in three types of mammalian cells (human lung epithelial carcinoma, human hepatocarcinoma and non-human primate Vero E6 cells) in response to infection with pathogenic Romero strain and no detectable mono- and oligo-nucleosome formation in Romero-infected Vero cells. The magnitude and kinetics of apoptosis induction in Huh7 and Vero cells were stronger upon infection with attenuated strain of JUNV. It seems conceivable that an induction of apoptosis upon Candid#1 infection in cells of mononuclear lineage, JUNV primary target [50]–[54] or parenchymal cells, may contribute to the host antiviral response by limiting virus replication and spread, as well as increasing clearance and immunogenicity of infected cells [46], [55], [56]. For instance, immunogenicity of apoptotic cancer cells has been attributed to the exposure of calreticulin, an endoplasmic reticulum chaperon, on the cell surface during early apoptosis. TLR4 on immature DCs recognizes calreticulin, stimulating antigen processing and presentation. The release of high-mobility group box 1 (HMGB1) chromatin-binding protein to the extracellular space during late apoptosis has the same effect [55], [56]. Moreover, mouse macrophages have been shown to specifically phagocytose apoptotic mouse thymocytes with PS on the outer leaflet of the plasma membrane [46]. At the same time, a pathogenic role of apoptosis induction in response to viral infections has been documented [57], [58]. In macaque, guinea pig and type I and II IFN receptor deficient mouse models of Argentine hemorrhagic fever multiple tangible body macrophages have been detected in spleen of infected animals [59]–[61]. Germinal center tingible body macrophages contain stainable condensed chromatin fragments of phagocytized, apoptotic cells [62]. Moreover, chromatolysis and pyknosis in neurons suggestive of neuronal apoptosis and/or necrosis was detected in a study of 10 autopsy cases of AHF [63]. These observations do not indicate that infected cells undergo apoptosis, however, they suggest a possible pathogenic role of apoptosis in JUNV infection.

IFN-I independent RLH-mediated induction of apoptosis in response to dsRNA, RNA and DNA viruses has been documented [35]–[39]. Likewise, deficiencies in RLH or apoptotic pathways often result in enhanced viral replication or pathogenicity in cultured cells and animal models [34], [39]. Accordingly, siRNA-mediated down-regulation of RIG-I and IRF3 expression increased viability of Candid#1-infected A549 cells despite enhanced viral production. Transient effect of siRNA knockdown and the ISG nature of RIG-I could have contributed to the moderate increase we observed in cell viability and virus production in infected cells. We also found drastically reduced DNA fragmentation in RIG-I deficient A549 RIG-I KD and Huh7.5 cells infected with JUNV relative to that of the corresponding infected RIG-I competent controls. Our data indicate that RIG-I contributes to induction of the programmed cell death in response to JUNV infection. Supporting type I IFN independent mechanism of apoptosis induction in response to JUNV infection, we detected DNA fragmentation in Candid#1- or Romero-infected type I IFN-deficient Vero or VeroE6 cells, respectively.

Our observation of detectable levels of DNA fragmentation in Romero-infected Vero E6 cells appears to contradict the recent report [23], which shows the lack of apoptosis in Romero virus infected Vero E6 cells. These seemingly conflicting findings may be related to the sensitivity of the assays used to evaluate apoptosis. In the aforementioned study the absence of apoptosis in Romero-infected Vero E6 cells was based on the lack of visible chromatin condensation by microscopic examination after DAPI staining and undetectable PARP/CASP3 cleavage as determined by western blotting [23]. In contrast, we have used an ELISA-based assay that allowed us to detect and quantify formation of mono- and oligo-nucleosome in low number of cells (assay detection limit is 1000 cells). These data illustrate the need of using several alternative and carefully characterized assays to study the interplay between JUNV infection and host apoptosis response.

In spite of conservation of proposed CASP cleavage motifs [23] in NP of Candid#1, infection with this virus induced cytoplasmic nucleosomes in Vero and human hepatocarcinoma cells. Additionally, we documented apoptosis induction in human lung epithelial carcinoma cells in response to Candid#1 infection using four different experimental approaches. Similarly, we detected pronounced DNA fragmentation in two human cell lines upon Romero infection. Only in Vero E6 cells we observed a lack of considerable apoptosis upon Candid#1-infection. Likewise, DNA fragmentation was significantly reduced in Romero-infected Vero E6 cells relative to that of Candid#1-infected Vero cells. These results suggest that absence/reduction of apoptosis induction in JUNV-infected Vero E6 cells could be a clone-specific phenomenon. It is possible that a host factor required for Candid#1 induced apoptosis is present in the majority of Vero cells, but absent in the sub-population of cells that were selected as Vero E6 clone. Similarly to our data, CPE in response to Middle East Respiratory Syndrome coronavirus infection was significantly delayed and less profound in Vero E6 cells compare with that of Vero cell [64]. Future studies of molecular defect(s) in Vero E6 should help to address the inability of these cells to undergo apoptosis upon infection with JUNV.

In summary, we have presented evidence of apoptosis induction in response to JUNV infection. Vaccine strain of JUNV Candid#1 induces more potent apoptosis than virulent Romero strain in human hepatocarcinoma and Vero cells, but not in A549 or Vero E6 cells. We have also shown that RIG-I, independent of type I IFN, enhances apoptosis in response to JUNV infection. Future studies should address the detailed molecular mechanism underlying JUNV induced apoptosis and its contribution to virus attenuation or pathogenesis.

## References

[1] Fields BN, Knipe DM, Howley PM (2013) Fields virology. Philadelphia: Wolters Kluwer Health/Lippincott Williams & Wilkins.

[2] Morin B, Coutard B, Lelke M, Ferron F, Kerber R, et al (2010) The N-terminal domain of the arenavirus L protein is an RNA endonuclease essential in mRNA transcription. PLoS Pathog 6: e1001038. 20862324 10.1371/journal.ppat.1001038 PMC2940758

[3] Perez M, Craven RC, de la Torre JC (2003) The small RING finger protein Z drives arenavirus budding: implications for antiviral strategies. Proc Natl Acad Sci U S A 100: 12978–12983. 14563923 10.1073/pnas.2133782100 PMC240730

[4] Hastie KM, Kimberlin CR, Zandonatti MA, MacRae IJ, Saphire EO (2011) Structure of the Lassa virus nucleoprotein reveals a dsRNA-specific 3′ to 5′ exonuclease activity essential for immune suppression. Proc Natl Acad Sci USA 108: 2396–2401. 21262835 10.1073/pnas.1016404108 PMC3038715

[5] Lenz O, ter Meulen J, Klenk HD, Seidah NG, Garten W (2001) The Lassa virus glycoprotein precursor GP-C is proteolytically processed by subtilase SKI-1/S1P. Proc Natl Acad Sci U S A 98: 12701–12705. 11606739 10.1073/pnas.221447598 PMC60117

[6] Charrel RN, de Lamballerie X, Emonet S (2008) Phylogeny of the genus Arenavirus. Curr Opin Microbiol 11: 362–368. 18602020 10.1016/j.mib.2008.06.001

[7] Briese T, Paweska JT, McMullan LK, Hutchison SK, Street C, et al (2009) Genetic detection and characterization of Lujo virus, a new hemorrhagic fever-associated arenavirus from southern Africa. PLoS Pathog 5: e1000455. 19478873 10.1371/journal.ppat.1000455 PMC2680969

[8] Charrel RN, de Lamballerie X (2010) Zoonotic aspects of arenavirus infections. Vet Microbiol 140: 213–220. 19748747 10.1016/j.vetmic.2009.08.027

[9] Harrison LH, Halsey NA, McKee KT Jr, Peters CJ, Barrera Oro JG, et al (1999) Clinical case definitions for Argentine hemorrhagic fever. Clin Infect Dis 28: 1091–1094. 10452640 10.1086/514749

[10] Maiztegui J, Feuillade M, Briggiler A (1986) Progressive extension of the endemic area and changing incidence of Argentine Hemorrhagic Fever. Med Microbiol Immunol 175: 149–152. 3014288 10.1007/BF02122437

[11] Maiztegui JI (1975) Clinical and epidemiological patterns of Argentine haemorrhagic fever. Bull World Health Organ 52: 567–575. 1085212 PMC2366633

[12] Bossi P, Tegnell A, Baka A, Van Loock F, Hendriks J, et al (2004) Bichat guidelines for the clinical management of haemorrhagic fever viruses and bioterrorism-related haemorrhagic fever viruses. Euro Surveill 9: E11–12. 15677844

[13] Kenyon RH, McKee KT Jr, Zack PM, Rippy MK, Vogel AP, et al (1992) Aerosol infection of rhesus macaques with Junin virus. Intervirology 33: 23–31. 1371270 10.1159/000150227

[14] Ruggiero HA, Perez Isquierdo F, Milani HA, Barri A, Val A, et al (1986) Treatment of Argentine hemorrhagic fever with convalescent’s plasma. 4433 cases. Presse Med 15: 2239–2242. 2949253

[15] Enria DA, Briggiler AM, Sanchez Z (2008) Treatment of Argentine hemorrhagic fever. Antiviral Res 78: 132–139. 18054395 10.1016/j.antiviral.2007.10.010 PMC7144853

[16] Enria DA, Briggiler AM, Levis S, Vallejos D, Maiztegui JI, et al (1987) Tolerance and antiviral effect of ribavirin in patients with Argentine hemorrhagic fever. Antiviral Res 7: 353–359. 2445283 10.1016/0166-3542(87)90017-9

[17] Enria DA, Maiztegui JI (1994) Antiviral treatment of Argentine hemorrhagic fever. Antiviral Res 23: 23–31. 8141590 10.1016/0166-3542(94)90030-2

[18] NIAID (2013) Biodefense and Emerging Infectious Diseases. NIAID Category A, B, and C Priority Pathogens.

[19] CDC (2000) Biological and Chemical Terrorism:Strategic Plan for Preparedness and Response. Recommendations of the CDC Strategic Planning Workgroup: Morbidity and Mortality Weekly Report 49(RR04): 1–14. 10803503

[20] Animal and Plant Health Inspection Service, CDC (nd) Select Agents and Toxins List.

[21] Muller S, Geffers R, Gunther S (2007) Analysis of gene expression in Lassa virus-infected HuH-7 cells. J Gen Virol 88: 1568–1575. 17412988 10.1099/vir.0.82529-0

[22] Groseth A, Hoenen T, Weber M, Wolff S, Herwig A, et al (2011) Tacaribe virus but not junin virus infection induces cytokine release from primary human monocytes and macrophages. PLoS Negl Trop Dis 5: e1137. 21572983 10.1371/journal.pntd.0001137 PMC3091837

[23] Wolff S, Becker S, Groseth A (2013) Cleavage of the Junin virus nucleoprotein serves a decoy function to inhibit the induction of apoptosis during infection. J Virol 87: 224–233. 23077297 10.1128/JVI.01929-12 PMC3536391

[24] Soares MM, King SW, Thorpe PE (2008) Targeting inside-out phosphatidylserine as a therapeutic strategy for viral diseases. Nat Med 14: 1357–1362. 19029986 10.1038/nm.1885 PMC2597367

[25] Maiztegui JI, McKee KT Jr, Barrera Oro JG, Harrison LH, Gibbs PH, et al (1998) Protective efficacy of a live attenuated vaccine against Argentine hemorrhagic fever. AHF Study Group. J Infect Dis 177: 277–283. 9466512 10.1086/514211

[26] Contigiani M, Medeot S, Diaz G (1993) Heterogeneity and stability characteristics of Candid 1 attenuated strain of Junin virus. Acta Virol 37: 41–46. 8105651

[27] Goni SE, Iserte JA, Stephan BI, Borio CS, Ghiringhelli PD, et al (2010) Molecular analysis of the virulence attenuation process in Junin virus vaccine genealogy. Virus Genes 40: 320–328. 20148301 10.1007/s11262-010-0450-2

[28] Goni SE, Iserte JA, Ambrosio AM, Romanowski V, Ghiringhelli PD, et al (2006) Genomic features of attenuated Junin virus vaccine strain candidate. Virus Genes 32: 37–41. 16525733 10.1007/s11262-005-5843-2

[29] Albarino CG, Bird BH, Chakrabarti AK, Dodd KA, Flint M, et al. The major determinant of attenuation in mice of the Candid1 vaccine for Argentine hemorrhagic fever is located in the G2 glycoprotein transmembrane domain. J Virol. 10.1128/JVI.00856-11 PMC3196416 21795336

[30] Albarino CG, Ghiringhelli PD, Posik DM, Lozano ME, Ambrosio AM, et al (1997) Molecular characterization of attenuated Junin virus strains. J Gen Virol 78 (Pt 7): 1605–1610. 10.1099/0022-1317-78-7-1605 9225036

[31] Garcia JB, Morzunov SP, Levis S, Rowe J, Calderon G, et al (2000) Genetic diversity of the Junin virus in Argentina: geographic and temporal patterns. Virology 272: 127–136. 10873755 10.1006/viro.2000.0345

[32] Ghiringhelli PD, Albarino CG, Piboul M, Romanowski V (1997) The glycoprotein precursor gene of the attenuated Junin virus vaccine strain (Candid #1). Am J Trop Med Hyg 56: 216–225. 9080883 10.4269/ajtmh.1997.56.216

[33] Huang C, Kolokoltsova OA, Yun NE, Seregin AV, Poussard AL, et al (2012) Junin virus infection activates the type I interferon pathway in a RIG-I-dependent manner. PLoS Negl Trop Dis 6: e1659. 22629479 10.1371/journal.pntd.0001659 PMC3358329

[34] Peters K, Chattopadhyay S, Sen GC (2008) IRF-3 activation by Sendai virus infection is required for cellular apoptosis and avoidance of persistence. J Virol 82: 3500–3508. 18216110 10.1128/JVI.02536-07 PMC2268502

[35] Lei Y, Moore CB, Liesman RM, O’Connor BP, Bergstralh DT, et al (2009) MAVS-mediated apoptosis and its inhibition by viral proteins. PLoS One 4: e5466. 19404494 10.1371/journal.pone.0005466 PMC2674933

[36] Eitz Ferrer P, Potthoff S, Kirschnek S, Gasteiger G, Kastenmuller W, et al (2011) Induction of Noxa-mediated apoptosis by modified vaccinia virus Ankara depends on viral recognition by cytosolic helicases, leading to IRF-3/IFN-beta-dependent induction of pro-apoptotic Noxa. PLoS Pathog 7: e1002083. 21698224 10.1371/journal.ppat.1002083 PMC3116819

[37] Vince JE (2010) Tschopp (2010) J IRF-3 partners Bax in a viral-induced dance macabre. Embo J 29: 1627–1628. 20485298 10.1038/emboj.2010.79 PMC2876974

[38] Chattopadhyay S, Marques JT, Yamashita M, Peters KL, Smith K, et al (2010) Viral apoptosis is induced by IRF-3-mediated activation of Bax. Embo J 29: 1762–1773. 20360684 10.1038/emboj.2010.50 PMC2876960

[39] Chattopadhyay S, Yamashita M, Zhang Y, Sen GC (2011) The IRF-3/Bax-mediated apoptotic pathway, activated by viral cytoplasmic RNA and DNA, inhibits virus replication. J Virol 85: 3708–3716. 21307205 10.1128/JVI.02133-10 PMC3126131

[40] Besch R, Poeck H, Hohenauer T, Senft D, Hacker G, et al (2009) Proapoptotic signaling induced by RIG-I and MDA-5 results in type I interferon-independent apoptosis in human melanoma cells. J Clin Invest 119: 2399–2411. 19620789 10.1172/JCI37155 PMC2719920

[41] Desmyter J, Melnick JL, Rawls WE (1968) Defectiveness of interferon production and of rubella virus interference in a line of African green monkey kidney cells (Vero). J Virol 2: 955–961. 4302013 10.1128/jvi.2.10.955-961.1968 PMC375423

[42] Park SH, Choi J, Kang JI, Choi SY, Hwang SB, et al (2006) Attenuated expression of interferon-induced protein kinase PKR in a simian cell devoid of type I interferons. Mol Cells 21: 21–28. 16511343

[43] Blight KJ, McKeating JA, Rice CM (2002) Highly permissive cell lines for subgenomic and genomic hepatitis C virus RNA replication. J Virol 76: 13001–13014. 12438626 10.1128/JVI.76.24.13001-13014.2002 PMC136668

[44] McKee KT Jr, Mahlandt BG, Maiztegui JI, Eddy GA, Peters CJ (1985) Experimental Argentine hemorrhagic fever in rhesus macaques: viral strain-dependent clinical response. J Infect Dis 152: 218–221. 2989384 10.1093/infdis/152.1.218

[45] U.S. Department of Health and Human Services, Centers for Disease Control Prevention, National Institutes of Health (2009) Biosafety in Microbiological and Biomedical Laboratories. 5th Edition ed. (December 2009).

[46] Fadok VA, Voelker DR, Campbell PA, Cohen JJ, Bratton DL, et al (1992) Exposure of phosphatidylserine on the surface of apoptotic lymphocytes triggers specific recognition and removal by macrophages. J Immunol 148: 2207–2216. 1545126

[47] Arends MJ, Morris RG, Wyllie AH (1990) Apoptosis. The role of the endonuclease. Am J Pathol 136: 593–608. 2156431 PMC1877493

[48] Sumpter R Jr, Loo YM, Foy E, Li K, Yoneyama M, et al (2005) Regulating intracellular antiviral defense and permissiveness to hepatitis C virus RNA replication through a cellular RNA helicase, RIG-I. J Virol 79: 2689–2699. 15708988 10.1128/JVI.79.5.2689-2699.2005 PMC548482

[49] Maher SG, Romero-Weaver AL, Scarzello AJ, Gamero AM (2007) Interferon: cellular executioner or white knight? Curr Med Chem 14: 1279–1289. 17504213 10.2174/092986707780597907

[50] Ambrosio M, Vallejos A, Saavedra C, Maiztegui JI (1990) Junin virus replication in peripheral blood mononuclear cells of patients with Argentine haemorrhagic fever. Acta Virol 34: 58–63. 1975726

[51] Gonzalez PH, Cossio PM, Arana R, Maiztegui JI, Laguens RP (1980) Lymphatic tissue in Argentine hemorrhagic fever. Pathologic features. Arch Pathol Lab Med 104: 250–254. 6154445

[52] Laguens M, Chambo JG, Laguens RP (1983) In vivo replication of pathogenic and attenuated strains of Junin virus in different cell populations of lymphatic tissue. Infect Immun 41: 1279–1283. 6309667 10.1128/iai.41.3.1279-1283.1983 PMC264636

[53] Carballal G, Cossio PM, Laguens RP, Ponzinibbio C, Oubina JR, et al (1981) Junin virus infection of guinea pigs: immunohistochemical and ultrastructural studies of hemopoietic tissue. J Infect Dis 143: 7–14. 6260868 10.1093/infdis/143.1.7

[54] Laguens RM, Chambo JG, Laguens RP (1986) Splenic dendritic cells and Junin virus. Med Microbiol Immunol 175: 187–189. 2425232 10.1007/BF02122447

[55] Kepp O, Tesniere A, Schlemmer F, Michaud M, Senovilla L, et al (2009) Immunogenic cell death modalities and their impact on cancer treatment. Apoptosis 14: 364–375. 19145485 10.1007/s10495-008-0303-9

[56] Kepp O, Tesniere A, Zitvogel L, Kroemer G (2009) The immunogenicity of tumor cell death. Curr Opin Oncol 21: 71–76. 19125021 10.1097/CCO.0b013e32831bc375

[57] Martins Sde T, Silveira GF, Alves LR, Duarte dos Santos CN, Bordignon J (2012) Dendritic cell apoptosis and the pathogenesis of dengue. Viruses 4: 2736–2753. 23202502 10.3390/v4112736 PMC3509670

[58] Licata JM, Harty RN (2003) Rhabdoviruses and apoptosis. Int Rev Immunol 22: 451–476. 12959754 10.1080/08830180305217

[59] Yun NE, Linde NS, Dziuba N, Zacks MA, Smith JN, et al (2008) Pathogenesis of XJ and Romero strains of Junin virus in two strains of guinea pigs. Am J Trop Med Hyg 79: 275–282. 18689636 PMC2700623

[60] Green DE, Mahlandt BG, McKee KT Jr (1987) Experimental Argentine hemorrhagic fever in rhesus macaques: virus-specific variations in pathology. J Med Virol 22: 113–133. 3039051 10.1002/jmv.1890220203

[61] Kolokoltsova OA, Yun NE, Poussard AL, Smith JK, Smith JN, et al (2010) Mice lacking alpha/beta and gamma interferon receptors are susceptible to junin virus infection. J Virol 84: 13063–13067. 20926559 10.1128/JVI.01389-10 PMC3004311

[62] Rahman ZS (2011) Impaired clearance of apoptotic cells in germinal centers: implications for loss of B cell tolerance and induction of autoimmunity. Immunol Res 51: 125–133. 22038528 10.1007/s12026-011-8248-4

[63] Gallardo F (1970) Argentine hemorrhagic fever. Anatomo-pathological findings in 10 necropsies. Medicina (B Aires): Suppl 1: 77–84. 5504271

[64] de Wilde AH, Raj VS, Oudshoorn D, Bestebroer TM, van Nieuwkoop S, et al (2013) MERS-coronavirus replication induces severe in vitro cytopathology and is strongly inhibited by cyclosporin A or interferon-alpha treatment. J Gen Virol 94: 1749–1760. 23620378 10.1099/vir.0.052910-0 PMC3749523

